# APOB codon 4311 polymorphism is associated with hepatitis C virus infection through altered lipid metabolism

**DOI:** 10.1186/s12876-018-0747-5

**Published:** 2018-01-30

**Authors:** Rie Harada, Masako Kimura, Yasushi Sato, Tatsuya Taniguchi, Tetsu Tomonari, Takahiro Tanaka, Hironori Tanaka, Naoki Muguruma, Hirohiko Shinomiya, Hirohito Honda, Issei Imoto, Masahiro Sogabe, Toshiya Okahisa, Tetsuji Takayama

**Affiliations:** 1 Department of Gastroenterology and Oncology, Tokushima University Graduate School of Biomedical Sciences, 3-18-15, Kuramoto-cho, Tokushima, 770-8503 Japan; 2 Department of Gastroenterology, Yoshinogawa Medical Center, Yoshinogawa, Tokushima, 776-8511 Japan; 3 Department of Internal Medicine, Tokushima Health Screening Center, 1-10-3, Kuramoto-cho, Tokushima, 770-0042 Japan; 4 Department of Human Genetics, Tokushima University Graduate School of Biomedical Sciences, 3-18-15, Kuramoto-cho, Tokushima, 770-8503 Japan; 5 Department of General Medicine and Community Health Science, Tokushima University Graduate School of Biomedical Sciences, 3-18-15, Kuramoto-cho, Tokushima, 770-8503 Japan

**Keywords:** Hepatitis C virus, Lipid metabolism, SNPs, ApoB

## Abstract

**Background:**

It has been reported that some single-nucleotide polymorphisms (SNPs) in lipid regulators such as apolipoproteins and cell surface molecules for hepatitis C virus (HCV) entry into hepatocytes are associated with HCV infection. However, it is unknown how HCV infection is affected by altered lipid metabolism resulting from the SNPs. We investigated the relationship between these SNPs and HCV infection status, and also analyzed the mechanism by which these SNPs mediate HCV infection via lipid metabolism alterations.

**Methods:**

Serum lipid and apolipoprotein profiles were tested in 158 HCV-positive and 220 HCV-negative subjects. We selected 22 SNPs in five lipid regulator genes which were related to HCV entry into hepatocytes and to lipid metabolism (*APOA1*, *APOB*, *SR-B1*, *LDLR*, and *APOE*), and their polymorphisms were analyzed using the PCR-sequence-specific oligonucleotide probe-Luminex method.

**Results:**

An *APOB* N4311S (g.41553a > g) SNP, rs1042034, was significantly associated with HCV positivity; the HCV positivity rate for the minor allele AA genotype was significantly higher than for genotype AG + GG (*P* = 0.016). Other SNPs except for APOB P2712L SNP rs676210, which is in linkage disequilibrium with rs1042034, showed no significant difference in genotype distribution. The serum level of low density lipoprotein-cholesterol (LDL-C) in the genotype AA group was significantly lower than in the genotype non-AA group (*P* = 0.032), whereas the triglyceride (TG) level was significantly higher (*P* = 0.007).

**Conclusion:**

An APOB SNP, rs1042034, is closely associated with HCV infection through lipid metabolism alteration. The minor allele AA genotype might contribute to facilitating serum LDL uptake into hepatocytes via LDLR by modifying their affinity and interaction and may have an influence on HCV infection by their entry to the liver through the LDLR.

## Background

Hepatitis C virus (HCV) infects approximately 185 million people worldwide [1]. HCV infection typically causes chronic liver disease and can lead to liver cirrhosis and hepatocellular carcinoma (HCC) [2]. Accumulating studies have revealed that the HCV life cycle is closely associated with host lipid metabolism.

It has been reported that HCV entry into hepatocytes occurs through cell surface molecules such as low density lipoprotein receptor (LDLR), scavenger receptor class B type 1 (SR-B1), claudin-1 (CLDN1), DC-SIGN, L-SIGN, and CD81. However, the details of the underlying mechanisms have not yet been clarified [3]. Among these molecules LDLR and SR-B1 also play a crucial role in lipid metabolism. Apolipoproteins are proteins on the surface of lipoprotein particles and stabilize the structure of lipoproteins. Apolipoprotein B (APOB) is one of the major apolipoproteins and it transfers lipoproteins such as chylomicron, very low density lipoprotein (VLDL), and low density lipoprotein (LDL) as a carrier. It is secreted from the liver as a component of the outer surface of VLDL particles, which are also produced in the liver, and circulates in blood, irreversibly associating with VLDL and LDL. APOB-100 serves as a ligand for LDLRs on the cell membrane of hepatocytes. Thus, the LDL particle enters into hepatocyte through the LDLR [4–6].

In the HCV life cycle, HCV replication begins in a membranous web on the endoplasmic reticulum membrane in hepatocytes, where VLDL assembly also occurs. Both HCV particles and VLDL particles are secreted from hepatocytes and circulate in blood as a complex referred to as a lipo-viral particle (LVP). Furthermore, APOB-100 and apolipoprotein E (APOE), which are referred to as VLDL-associated proteins, are present on the surface of the complex. They form LVP with HCV particles, indicating that they are deeply involved in the formation of infectious HCV particles [7]. After circulation in the blood, infectious HCV particles enter into hepatocytes with LDL through the LDLR [8].

It is well known that apolipoproteins and cell surface molecules such as LDLR and SR-B1 have single-nucleotide polymorphisms (SNPs), and some of the SNPs cause lipid metabolism disorders, leading to a high risk of cardiovascular events [9–11]. Regarding the association with HCV, several studies have suggested that SNPs of these lipid-metabolic regulators correlate with HCV infection; e.g., an SNP in *LDLR* exon 13 (rs5925) is associated with susceptibility to HCV infection [12]; an SNP in *LDLR* exon8 (rs11669576) is associated with severity of hepatic fibrosis; and an SNP in *LDLR* exon10 (rs5930) is associated with viral clearance and overall inflammation of the liver [13]. SNPs in *LDLR* 3’UTR region (rs1433099) and intron 14 (rs2569540) have been reported to have an association with hepatitis C viral load in the pre-treatment state [14]. Furthermore, polymorphisms of *APOE* have been reported to be associated with persistent HCV infection and the degree of liver damage [15, 16].

Thus, it is assumed that SNPs of lipid regulators have a strict association with HCV infection and lipid metabolism disorders. However, the relationship between HCV infection and lipid metabolism disorder among the SNPs of these lipid regulators have not yet been fully elucidated. Therefore, in this study, we first investigated the relationship between the SNPs of lipid regulator genes such as apolipoproteins and cell surface molecules that affect the HCV life cycle, and HCV infection status comparing HCV-positive and HCV-negative subjects. We then examined differences in lipid metabolism between each SNP genotype and analyzed the causal relationship between the SNP and HCV infection status through altered lipid metabolism.

## Methods

### Subjects

This study included a total of 378 subjects who visited our university hospital and affiliated hospitals from November 2010 to March 2014. All subjects were Japanese and were between 41 and 76 years old. Patients who had received prior or ongoing anti-viral therapy and treatment for lipid metabolism disorders were excluded. We also excluded patients with inherited lipid metabolism disorders such as familial hypercholesterolemia and secondary lipid metabolism disorders such as hypothyroidism and kidney diseases.

An HCV antibody test was carried out for all subjects; 158 were HCV positive and HCV RNA detectable by RT-PCR, and 220 were HCV negative. Serum HCV RNA levels were measured using COBAS TaqMan HCV Auto Assay System (Roche Diagnostics, Tokyo, Japan; lower limit of quantification, 1.2 log IU/ml). HCV genotypes were determined by direct sequence method at BML, Inc. (Saitama, Japan). Serum transaminase, total bilirubin, albumin levels, and prothrombin time were measured, and ultrasonography or contrast-enhanced computed tomography was performed to check liver function and search for HCC. Based on these results, subjects with severe liver dysfunction of Child-Pugh class B and C were excluded from the study. Patients with conditions associated with liver dysfunction such as HCC, hepatitis B virus (HBV) infection, or nonalcoholic steatohepatitis (NASH) were also excluded from the study. The study protocol was approved by the institutional review board of each hospital and all patients provided written informed consent to participate in this study.

### Measurement of serum lipids and apolipoproteins

Serum lipids and apolipoproteins were tested after fasting overnight for 10 to 12 h. We measured low density lipoprotein-cholesterol (LDL-C), high density lipoprotein-cholesterol (HDL-C), and triglyceride (TG) according to standard procedures at our university hospital and affiliated hospitals. Serum levels of apolipoproteins (apolipoprotein A1 [APOA1], apolipoprotein A2 [APOA2], APOB, apolipoprotein C2 [APOC2], apolipoprotein C3 [APOC3], and APOE) were measured by immune nephelometry at SRL, Inc. (Tokyo, Japan).

### SNP selection

We selected 22 SNPs in five genes (*APOA1*, *APOB*, *SR-B1*, *LDLR*, and *APOE*) which are related to HCV entry into hepatocytes and are associated with lipid metabolism disorders according to previous studies; three SNPs (rs613808, rs2727784, and rs11216158) in *APOA1* [17], five SNPs (rs676210, rs1042031, rs693, rs1801701, and rs1042034) in *APOB* [9], three SNPs (rs4238001, rs61932577, and rs5888) in *SR-B1* [10], nine SNPs (rs17249134, rs17249141, rs2228671, rs11669576, rs5930, rs688, rs5925, rs2569542, and rs14158) in *LDLR* [13], and two SNPs (rs429358 and rs7412) in *APOE* [18].

### Genotyping

Genomic DNA was extracted from peripheral blood using the phenol-chloroform method and stored at − 4 °C. Genotypes of all SNPs selected in this study were analyzed at G&G Science Co., Ltd. (Fukushima, Japan) using the PCR-sequence-specific oligonucleotide probe-Luminex method, as described previously [19].

### Statistical analysis

Student’s *t*-test was used to compare serum lipid and apolipoprotein levels between the two subject groups (HCV-positive and HCV-negative) as well as the genotype groups for each SNP. The genotype distributions for each SNP were analyzed using *χ*^*2*^ test. A *P* value of less than 0.05 was considered statistically significant. The statistical analyses were performed using Statcel 4 (OMS Ltd., Saitama, Japan). Linkage disequilibrium was analyzed using the program Haploview from the Broad Institute website (https://www.broadinstitute.org/haploview/haploview).

## Results

### Characteristics of the subjects

The study included 158 patients with HCV infection and 220 subjects without HCV infection. The age and sex ratio were well balanced between the groups. There was no significant difference in serum total bilirubin, AST, ALT and albumin levels, and prothrombin values (%), which are indicative of hepatic functional reserve, and were also well balanced between groups. HCV infected patients were genotype 1b (*n* = 128), 2a (*n* = 5), 2b (*n* = 9), 3a (*n* = 1), and undetermined (*n* = 6). The remaining nine patients received only serotype analysis: serotype I (*n* = 7) and serotype II (*n* = 2).

Among 158 subjects with HCV infection, the estimated median duration of HCV infection was 51 (IQR 44.3–55.0) years, and the median HCV RNA concentration in HCV-infected patients was 6.5 (IQR 6.0–6.9) log IU/ml.

### Profile of serum lipids and apolipoproteins in HCV-positive and HCV-negative subjects

Since HCV infection reportedly causes a change in serum lipid levels, we first compared the serum lipid levels between 158 HCV-positive subjects and 220 HCV-negative subjects (Table 1). The serum levels of total cholesterol (TC), LDL-C, and HDL-C in HCV-positive subjects were significantly lower than in HCV-negative subjects, whereas the serum TG level in HCV-positive subjects was higher than in HCV-negative subjects, although the difference was not statistically significant. Moreover, serum levels of all six apolipoproteins (APOA1, APOA2, APOB, APOC2, APOC3, and APOE) in HCV-positive subjects were significantly lower than in HCV-negative subjects. These results were fairly consistent with previous reports [6, 20, 21].

**Table 1:** Profile of serum lipids and apolipoproteins in HCV-positive and HCV-negative subjects

		Normal range	HCV+	HCV-	*P*-value
TC	(mg/dl)	120.0–219.0	169.4 ± 31.3	207.2 ± 32.0	< 0.001
LDL-C	(mg/dl)	65.0–139.0	96.8 ± 24.3	124.8 ± 29.5	< 0.001
HDL-C	(mg/dl)	40.0–90.0	53.1 ± 18.5	62.9 ± 17.6	< 0.001
TG	(mg/dl)	30.0–149.0	106.6 ± 52.2	98.3 ± 56.6	0.173
APOA1	(mg/dl)	120.0–160.0	139.6 ± 31.3	155.2 ± 29.2	< 0.001
APOA2	(mg/dl)	25.0–34.6	29.6 ± 5.5	30.8 ± 4.9	0.022
APOB	(mg/dl)	70.0–106.0	77.2 ± 17.9	96.7 ± 22.8	< 0.001
APOC2	(mg/dl)	1.6–4.2	2.1 ± 1.0	4.0 ± 1.7	< 0.001
APOC3	(mg/dl)	5.6–9.5	6.0 ± 2.3	9.2 ± 3.1	< 0.001
APOE	(mg/dl)	2.7–4.5	4.4 ± 1.1	4.7 ± 1.2	0.011

Data represent mean and standard deviation values and *P*-values were calculated using Student’s t-test

### Association of SNP genotype with HCV infection

To examine the relationship between SNP genotype in lipid regulator genes and HCV infection status, we analyzed the genotype distribution of the selected 22 SNPs in *APOA1*, *APOB*, *SR-B1*, *LDLR*, and *APOE*, and compared them between HCV-positive and HCV-negative groups (Table 2). Of the 22 SNPs examined, five were monomorphic in our study population; rs1801701 in *APOB*; rs61932577 in *SR-B1*; rs17249134, rs17249141, and rs11669576 in *LDLR*.

**Table 2:** Association of SNP genotype with HCV infection

		Alleles	HCV	Overall genotype distribution(n)			*P*-value	Comparison of specific genotypes	*P*-value
							(χ2 value)		(χ2 value)
APOA1	rs613808	G/A		AA	GA	GG			
			+	72	74	12	0.192		
			–	120	83	17	(3.297)		
	rs2727784	G/A		GG	GA	AA			
			+	75	71	12	0.290		
			–	122	82	16	(2.473)		
	rs11216158	A/G		GG	AG	AA			
			+	99	56	3	0.231		
			–	150	62	8	(2.933)		
APOB	rs676210	C/T		TT	CT	CC			
			+	88	57	13	0.091	TT + CT vs CC	0.031
			–	133	80	7	(4.784)		(4.673)
	rs1042031	G/A		GG	GA	AA			
			+	150	8	0			
			–	205	15	0			
	rs693	C/T		CC	CT	TT			
			+	147	11	0			
			–	199	21	0			
	rs1801701	G/A		GG	GA	AA			
			+	158	0	0			
			–	220	0	0			
	rs1042034	A/G		GG	AG	AA			
			+	88	57	13	0.051	GG + AG vs AA	0.016
			–	134	80	6	(5.963)		(5.828)
SR-B1	rs4238001	G/A		GG	GA	AA			
			+	157	1	0			
			–	220	0	0			
	rs61932577	C/T		CC	CT	TT			
			+	158	0	0			
			–	220	0	0			
	rs5888	C/T		CC	CT	TT			
			+	96	54	10	0.761		
			–	128	82	10	(0.546)		
LDLR	rs17249134	T/G		GG	TG	TT			
			+	158	0	0			
			–	220	0	0			
	rs17249141	T/C		CC	TC	TT			
			+	158	0	0			
			–	220	0	0			
	rs2228671	C/T		CC	CT	TT			
			+	156	2	0			
			–	219	1	0			
	rs11669576	A/G		GG	AG	AA			
			+	158	0	0			
			–	220	0	0			
	rs5930	G/A		GG	GA	AA			
			+	66	73	19	0.957		
			–	89	105	26	(0.088)		
	rs688	T/C		CC	TC	TT			
			+	120	36	2	0.664		
			–	160	55	5	(0.820)		
	rs5925	C/T		TT	CT	CC			
			+	114	42	2	0.479		
			–	149	65	6	(1.472)		
	rs2569542	A/G		GG	AG	AA			
			+	138	19	1	0.935		
			–	190	28	2	(0.135)		
	rs14158	G/A		GG	GA	AA			
			+	58	75	25	0.928		
			–	84	100	36	(0.150)		
APOE	rs429358	T/C		TT	TC	CC			
			+	131	27	0	0.564		
			–	177	41	2	(0.333)		
	rs7412	C/T		CC	CT	TT			
			+	142	16	0			
			–	196	24	0			

In the comparison of overall genotype distributions for each SNP, an *APOB* N4311S (g.41553a > g) SNP, rs1042034, showed a marginally non-significant trend with respect to HCV positivity (*P* = 0.051, *χ*^*2*^ = 5.963); the frequency of the A allele in HCV-positive subjects was higher than in HCV-negative subjects. In the comparison of specific genotypes (GG + AG vs. AA), the HCV positivity rate in the genotype AA group was significantly higher than in the genotype GG + AG group (*P* = 0.016, *χ*^*2*^ = 5.828). These results suggest that minor allele homozygosity (genotype AA) in rs1042034 might be associated with HCV infection. Moreover, an *APOB* P2712L (g.35310c > t) SNP, rs676210, showed a similar tendency to that of rs1042034; i.e., the frequency of the genotype CC allele in rs676210 was significantly higher than that of genotype TT + CT in HCV-positive subjects (*P* = 0.031, *χ*^*2*^ = 4.673). Other SNPs aside from these two showed no significant difference in genotype distribution between HCV-positive and HCV-negative subjects.

The rs1042034 SNP is located at the *APOB* carboxyl-terminal site, 6243 nucleotides away from rs676210, and this is reportedly in strong linkage disequilibrium [9]. The degree of linkage disequilibrium between these SNPs in our study population was also strong (D’ = 1, *r*^2^ = 0.98). Therefore, we confined the following association analysis of these SNPs with serum lipids only to rs1042034.

### Association of serum lipid and apolipoprotein levels with the rs1042034 genotype

Since APOB is reported to be associated with serum lipid levels [9], we compared the serum lipid levels between genotypes AA and non-AA (GG + AG) in rs1042034 (Table 3). The serum LDL-C level in the genotype AA group was significantly lower than that in the genotype non-AA group (98.7 ± 39.4 vs. 115.1 ± 30.1, *P* = 0.032), whereas the TG level was significantly higher in the genotype AA group than in the genotype non-AA group (137.7 ± 67.7 vs. 100.7 ± 55.3, *P* = 0.007). There were no significant differences in serum levels of APOA1, APOA2, APOB, APOC2, APOC3, and APOE.

**Table 3:** Association of serum lipid and apolipoprotein levels with the rs1042034 genotype

		Genotype of rs1042034		*P*-value
		AA (n)	GG + AG (n)	
TC	(mg/dl)	176.7 ± 46.5 (17)	191.8 ± 36.2 (332)	0.100
LDL-C	(mg/dl)	98.7 ± 39.4 (17)	115.1 ± 30.1 (316)	0.032
HDL-C	(mg/dl)	50.8 ± 19.4 (15)	59.6 ± 18.4 (302)	0.074
TG	(mg/dl)	137.7 ± 67.7 (18)	100.7 ± 55.3 (321)	0.007
APOA1	(mg/dl)	137.5 ± 34.1 (19)	149.3 ± 30.9 (355)	0.105
APOA2	(mg/dl)	29.6 ± 4.9 (19)	30.4 ± 5.2 (355)	0.547
APOB	(mg/dl)	83.6 ± 29.5 (19)	88.9 ± 22.6 (355)	0.335
APOC2	(mg/dl)	3.2 ± 1.9 (19)	3.2 ± 1.7 (355)	0.945
APOC3	(mg/dl)	7.9 ± 3.6 (19)	7.9 ± 3.2 (355)	0.975
APOE	(mg/dl)	4.8 ± 1.9 (19)	4.5 ± 1.1 (355)	0.390

Values are expressed as mean ± standard deviation. The number of subjects available for the analysis is shown in parentheses. *P*-values were calculated using Student’s t-test

To assess the effect of HCV status on lipid levels, we compared serum lipid and apolipoprotein levels between AA and non-AA genotypes of rs1042034 in HCV-positive and HCV-negative subjects (Table 4). No significant differences were observed in the levels of apolipoproteins between the AA and non-AA groups regardless of HCV status.

**Table 4:** Associations of serum lipid and apolipoprotein levels with rs1042034 genotype in HCV-positive and HCV-negative subjects

		HCV	Genotype of rs1042034		*P*-value
			AA (n)	GG + AG (n)	
TC	(mg/dl)	+	160.9 ± 43.5 (11)	170.3 ± 30.1 (138)	0.342
		–	205.7 ± 39.7 (6)	207.1 ± 32.0 (194)	0.917
LDL-C	(mg/dl)	+	86.2 ± 31.9 (11)	97.7 ± 23.2 (114)	0.134
		–	121.5 ± 44.4 (6)	124.9 ± 29.1 (202)	0.779
HDL-C	(mg/dl)	+	47.1 ± 22.6 (9)	53.6 ± 18.2 (110)	0.313
		–	56.5 ± 13.1 (6)	63.1 ± 17.7 (192)	0.370
TG	(mg/dl)	+	135.3 ± 66.1 (12)	106.3 ± 54.8 (127)	0.088
		–	142.5 ± 31.5 (6)	97.0 ± 55.4 (194)	0.052
APOA1	(mg/dl)	+	130.6 ± 35.8 (13)	140.5 ± 31.1 (144)	0.278
		–	152.3 ± 27.0 (6)	155.3 ± 29.3 (211)	0.805
APOA2	(mg/dl)	+	29.9 ± 5.6 (13)	29.6 ± 5.5 (144)	0.868
		–	29.1 ± 3.5 (6)	30.9 ± 4.9 (211)	0.374
APOB	(mg/dl)	+	74.1 ± 21.8 (13)	77.7 ± 17.6 (144)	0.490
		–	104.3 ± 35.3 (6)	96.5 ± 22.4 (211)	0.407
APOC2	(mg/dl)	+	2.6 ± 1.3 (13)	2.1 ± 1.0 (144)	0.056
		–	4.5 ± 2.6 (6)	4.0 ± 1.6 (211)	0.505
APOC3	(mg/dl)	+	6.6 ± 2.7 (13)	6.0 ± 2.3 (144)	0.394
		–	10.7 ± 3.9 (6)	9.1 ± 3.0 (211)	0.210
APOE	(mg/dl)	+	4.8 ± 2.0 (13)	4.3 ± 1.0 (144)	0.114
		–	4.7 ± 1.7 (6)	4.7 ± 1.2 (211)	0.979

Values are presented as mean ± standard deviation. The number of subjects available for the analysis is shown in parentheses. *P*-values were calculated using Student‘s t-test

## Discussion

In this study, we demonstrated that the AA genotype of rs1042034 in the APOB gene is significantly related to HCV infection status and might be associated with the regulation of serum LDL-C and TG levels. These results could suggest a close association between HCV infection status and lipid metabolism alterations due to the APOB SNP.

HCV exists in blood as an LVP, with which APOB and APOE are also combined [22]. HCV in LVPs can enter into hepatocytes through LDLRs on the cell membrane [23]. APOB-100 is deeply involved in these steps as a ligand for the LDLR; however, the underlying mechanisms have not yet been clarified in detail [24]. Previously, Li and colleagues reported that an SNP in *LDLR* exon 13 (rs5925) was associated with susceptibility to HCV infection [12], but we did not detect a significant difference in the SNP in our cohort. This difference may be due to the fact that the previous study involved a smaller subject cohort (a total of 156 subjects) as compared with our study (378 subjects). Zhu and associates reported that an SNP in the *APOB* promoter region (rs934197) was associated with susceptibility to HCV infection, and showed that HCV-infected patients had significantly lower levels of LDL-C as compared with healthy individuals [25]. Although we did not analyze SNPs in the promoter region, a similar tendency of lower LDL-C levels was observed; HCV-positive subjects with AA genotype of *APOB* exhibited lower LDL-C levels than the other genotypes. Moreover, rs1042034 in *APOB* is known to regulate biding to the LDLR and is therefore assumed to cause a change in the binding affinity of APOB to LDLRs [26]. These findings are further supportive of a close relationship between the life cycle of HCV and host *APOB* polymorphism.

Since serum lipid levels are very important risk factors for cardiovascular disease, several large-scale screenings for SNPs concerning serum lipid-regulator genes have been reported. Regarding studies of the *APOB* SNP rs1042034, the genome-wide association study (GWAS), which included more than 100,000 individuals of European ancestry, reported that polymorphism of rs1042034 was significantly associated with decreased TG and increased HDL-C levels [27]. In a study involving several ethnic groups, rs1042034 was significantly associated with lower TC and LDL-C levels only in Hispanics [28]. As for Asians, rs1042034 was significantly associated with lower TG and higher HDL-C levels in South Asians, compatible with the European GWAS, but there was no significant association in East Asians [27]. Although the underlying mechanism of the changes in serum lipid levels caused by this SNP have still not been elucidated, it appears *APOB-100* mutations and polymorphisms may change the conformation of APOB-100, thereby leading to a change in LDL metabolism through the interaction of APOB-100 with the LDLR.

Based on the findings mentioned above, we can speculate that a relationship exists between HCV infection and lipid metabolism disorders through SNPs of APOB-100, whereby the AA genotype (minor allele homozygote) of rs1042034 could facilitate serum LDL uptake via LDLR on hepatocytes by modifying their mutual affinity and interaction, and consequently, the serum LDL-C level could be decreased. As a result, the susceptibility to HCV infection could be increased because HCV particles exist as LVPs and enter into the liver with LDL. However, it is not clear why genotype AA was associated with higher serum TG levels as compared with genotype non-AA, and further studies are needed to investigate this relationship in greater detail.

In the comparison of lipid levels by genotype, serum levels of LDL-C and TG showed significant differences between AA and non-AA genotype (Table 3), whereas these differences disappeared when comparing by HCV status (Table 4), indicating that HCV infection itself did not have much influence on lipid metabolism, which supports our hypothesis.

Interestingly, a large Japanese cohort study revealed that HCV infection causes not only lower TC and LDL-C levels but also significant lower serum HDL-C and TG levels [21] and they suggest that the HCV infection itself might directly cause hypolipidemia, irrespective of host factors including age, hepatic damage, and nutritional state. Furthermore, it has been reported that HCV genotype 3a and 4 are associated with hypocholesterolemia in western patients [29, 30]. However, there was no association between HCV genotypes and lipid profile or SNP pattern in our cohort (data not shown). This may be partly explained by the fact that the most frequent genotype was 1b (81%), which is the most common genotype in the Japanese population, while genotypes 3a and 4 are very rare.

Therefore, the possibility of a direct interaction of HCV with lipid metabolism could not be completely ruled out in our study and need to be confirmed in a more detailed investigation.

There are some potential limitations of our study. First, regarding the HCV negative cohort, we could not completely rule out the possibility of including individuals in whom HCV had been eradicated naturally because we enrolled subjects who were negative for serum anti-HCV antibody. Second, the lipid metabolism profiles could have been altered by differences in liver function, which can be affected by various hepatic disorders*.* However, in the present study, we carefully limited the participants to Child-Pugh A, and excluded subjects with NASH, HBV infection, or HCC, and therefore the baseline characteristics and liver function levels of the participants were well balanced between the two groups. Although our study had a limitation in terms of not covering all SNPs in lipid regulators, our results suggested the possibility that an *APOB-100* SNP, rs1042034, influences the susceptibility to HCV infection by their entry into the liver through the LDLR. Our data may have important implications for identifying individuals with higher susceptibility to HCV infection via SNP of lipid regulators. Exploring the molecular mechanism underlying these results could provide important information for controlling the process of HCV infection and limiting the expansion of HCV infection.

In addition, in the treatment of chronic HCV infection, the rate of sustained virological response (SVR) subsequent to the approval of direct-acting antiviral agents has been dramatically increasing and the change in serum lipid levels after SVR is gaining attention [31]. Therefore, focusing on the influence of SNPs concerning lipid metabolism in HCV patients should have important implications for antivirus therapy in the future.

## Conclusions

In conclusion, this study demonstrated that an APOB SNP, rs1042034, is closely associated with HCV infection through lipid metabolism alteration. The minor allele AA genotype may facilitate serum LDL uptake into hepatocytes via LDLR by modifying their affinity and interaction and may have an influence on HCV infection by their entry into the liver through the LDLR. Further studies are needed to explore the mechanism underlying these results, which could provide important information for controlling the process of HCV infection.

## Abbreviations

APOA1: Apolipoprotein A1
APOA2: Apolipoprotein A2
APOB: Apolipoprotein B
APOC2: Apolipoprotein C2
APOC3: Apolipoprotein C3
APOE: Apolipoprotein E
HCV: Hepatitis virus C
HDL-C: High density lipoprotein cholesterol
LDL-C: Low density lipoprotein cholesterol
TC: Total cholesterol
TG: Total triglycerides
